# Improving phenylalanine and micronutrients status of children with phenylketonuria: a pilot randomized study

**DOI:** 10.1186/s13023-021-02094-8

**Published:** 2021-11-12

**Authors:** Reza Zamani, Akram Karimi-Shahanjarini, Leili Tapak, Babak Moeini

**Affiliations:** 1 Department of Public Health, School of Public Health, Hamadan University of Medical Sciences, Shaheed Fahmideh Ave., Hamadan, Iran; 2 Social Determinants of Health Research Center, Hamadan University of Medical Sciences, Hamadan, Iran; 3 Department of Biostatistics, School of Public Health, Modeling of Noncommunicable Diseases Research Center, Hamadan University of Medical Sciences, Hamadan, Iran

**Keywords:** Children, Micronutrients, Nutritional Status, Phenylalanine, Phenylketonuria

## Abstract

**Background:**

Children with Phenylketonuria (PKU) need a special diet to avoid a variety of physical and psychological complications. The aim of this study was to compare and assess the effects of two interventions on and levels of phenylalanine and micronutrients in children with PKU.

**Methods:**

Forty-six children with PKU (ages 1–12 years) and their caregivers were randomly assigned to one of two 10-week interventions: a caregiver educational intervention based on the Integrative Model of Behvioral Prediction (IMBP) and supplementary low-protein-modified foods. Outcomes consisted of Children's plasma phenylalanine and micronutrients (i.e., vitamin B12, vitamin D3, and zinc) and hemoglobin levels. To assess the sustainability of outcomes, we also compared the children’s phenylalanine level at five-time points including baseline and 10-week, 6 months, 12 months, and 24 months follow-ups. In addition, caregivers of both groups were asked to complete a questionnaire to assess how well the interventions were implemented as well as satisfaction with interventions.

**Results:**

While a large percentage of children had phenylalanine levels out of range indicating non-adherence (69.6% and 81% in the educational and food items group, respectively), micronutrient deficiencies were not prevalent in the patient cohort. The levels of phenylalanine in both groups decreased significantly over time. However, conducting a repeated-measures ANOVA to evaluating the change in groups across five-time points, revealed a significant difference between groups (F = 4.68, p = 0.03). That is, the educational intervention was more effective in lowering the children's phenylalanine level. At 24-month follow-up, the percentage of children with a normal range of phenylalanine level in the educational and food items groups increased to 73.9 and 57.1 percent, respectively, from 26 and 38 percent at baseline. There were no significant changes in children's micronutrients level following the interventions, except in the hemoglobin. In this way, at 10-week follow-up, the mean hemoglobin of children in the educational group reduced significantly (P = 0.041). However, there was no significant difference between the two groups. In general, all caregivers completed the process evaluation checklist, the feedback was largely positive.

**Conclusions:**

The results of this study demonstrated that both educational and providing food item interventions resulted in a significant reduction in phenylalanine levels. Empowering caregivers of patients, creating and fortifying social networks, providing favorable social supports, and providing access to special food items may be effective in controlling PKU.

*Clinical trial registration*: *Iranian Registry of Clinical Trials* (IRCT20180506039548N1). Registered 6th Jun 2018, https://www.irct.ir/trial/30977.

**Supplementary Information:**

The online version contains supplementary material available at 10.1186/s13023-021-02094-8.

## Introduction

Phenylketonuria (PKU) is an autosomal recessive disease that results in decreased metabolism of the amino acid phenylalanine. If left untreated, PKU is associated with severe intellectual disability. In countries without a neonatal screening program, PKU is one of the most common causes of mental retardation in children [1, 2]. The global prevalence of PKU is1:23,930 live births, however, there is a considerable variation across ethnicities and geographic regions worldwide [3]. A recent meta-analysis has estimated the prevalence of classical PKU to be 4.4/100,000 (95% CI 2.5–7.8) in Iranian newborns [4].

Treatment for PKU is based on dietary phenylalanine management consisting of taking a low-phenylalanine diet and a protein substitute that is free of or low in phenylalanine, as well as monitoring phenylalanine levels [5]. Despite the effectiveness of diet management and ongoing improvement of PKU formula and supplements, maintaining a regular and consistent diet is difficult [6] because of barriers such as limited access to low-protein modified foods as well as caregivers' poor knowledge and skills regarding PKU diet. Due to the restricted diet, nonadherence to the diet and lack of balance in taking various nutrients children with PKU may suffer from a deficiency in micronutrients such as vitamin D, iron, zinc, vitamin B12, and selenium [7–10]. Although low-protein foods are available, many families cannot afford them, which may contribute to an increase in patients' blood phenylalanine levels [11]. There is evidence supporting the effectiveness of some subsidy-based strategies in improving nutrition quality and reducing food insecurity among children [12]. To our knowledge, however, no study investigated the effectiveness of this form of intervention in controlling phenylalanine and micronutrients in PKU patients. On the other hand, while the studies indicate that family support, nutritional counseling for parents and caregivers, and their active involvement in training may improve the dietary adherence in children with a special need diet[6, 13–16] few studies have looked into the impact of interventions on dietary adherence in children with PKU. In assessing interventions of caregiver participation in children’s diet change, some conflicting results have been reported. For example, in the review by Niemeier et al., caregiver involvement was found to be successful [17]; however, some other evidence does not support the effectiveness of this approach [18]. These results can reflect the effect of using different techniques of behavior change, disease, and health status of children, age of children, and type of children's disease in need of a specific diet.

Evidence suggests that determinants such as the caregivers' knowledge and attitude towards disease and diet, ability to read food labels, and the ability to estimate the phenylalanine exchanges by eye may be useful in predicting diet adherence in caregivers [6, 19], so it appears that focusing on the variables suggested in the theoretical framework of the Integrative Model of Behavioral Prediction IMBP) could be beneficial in improving diet PKU adherence. Similar to the Theory of Planned Behavior (TPB), IMBP assumes that intention is the most important predictor of behavior. According to this framework, attitude, subjective norms, self-efficacy formed the individual’s intention, and supportive environment and skills—as actual control variables—moderate the relationship between intention and behavior [20]. Despite the advantage of IM, the majority of IMBP research does not use the framework completely. Dai reported that only in limited studies informed by IMBP, actual control variables (i.e., environment and skills) had been measured [21].The majority of PKU research has so far focused on determining the disease’s prevalence, genetic basis, and potential therapeutic options. Only a few studies have been conducted to evaluate the effectiveness of interventions of caregivers in diet modification for children with PKU. This study aimed to compare the effects of two interventions, an educational intervention based on the IMBP for the caregivers and providing specific food items, on phenylalanine levels and micronutrients in children with PKU.

## Methods

An equivalence trial was conducted to compare the effectiveness of two interventions, a caregiver educational intervention based on the integrative model of behavioral prediction (IM) and supplementary low-protein modified foods, on phenylalanine and micronutrients status of children with PKU. The children who took part in the study were those who had been diagnosed via newborn screening. The PKU screening program in Iran began in 2006. Within this program, following the detection of children with PKU, a regular follow-up is performed. Since PKU is a rare disease, no sampling was done and all children with PKU who were detected in Hamadan Province, Iran, were assessed for eligibility. Participants consisted of parents/ caregivers of 46 children with PKU and their parents/ caregivers. Inclusion criteria of caregivers included having a child of 1–12 years with a definitive diagnosis of mild PKU or classic type. We recruited this group of participants because adolescents have an increased desire for autonomy and tend to make decisions based on what their peers think, so their parents/ caregivers have less control over their diet. Some evidence suggests that caring interventions are more effective in caregivers of young children [22]. Children with malignant phenylketonuria were excluded from the study. As a result, 46 children with PKU and their caregivers participated in this study out of a total of 51. The written informed consent was obtained from participated caregivers. Eligible participants were randomized 1:1 to the educational intervention group, or the food items group by the permutation block method. The outcome consisted of children's phenylalanine and micronutrients levels.The study protocol was approved by the Ethics Committee and the Research Council at the authors' affiliated university.

### Measurements

Socio-demographics data were collected through interviews with caregivers (Table 1). Children’s plasma phenylalanine and vitamin B12, 25-hydroxy vitamin D, zinc, hemoglobin, and hematocrit levels were measured as outcomes. To quantify the plasma phenylalanine level at baseline, we used the mean of three measures from the children's clinical records throughout a 12-month period. Measuring phenylalanine level at 10 week-follow-up and micronutrients levels at baseline and a 10-week follow-up was done through obtaining children’s plasma samples. The samples were analyzed in the Be'sat hospital's laboratory where the PKU clinic is located in. We also gathered phenylalanine levels from children's clinical records at 6 months, 12 months, and 24 months after the intervention to assess the sustainability of results.

**Table 1:** Baseline characteristics (n = 44)

Variable	Food items group	Educational group	p-value
Patient age (mean of year)	6.76 ± 4.05	6.56 ± 3.78	0.869
Father's age (mean of year)	39.85 ± 7.55	40.08 ± 7.66	0.921
Mother's age (mean of year)	34.19 ± 7.60	35.26 ± 7.44	0.640
Number of family members (mean)	4.09 ± 0.99	4.34 ± 1.46	0.511
Number of child with PKU in the family	1.14 ± 0.35	1.08 ± 0.41	0.638
*Patient gender (%)*			0.222
Male	13 (61.9%)	10 (43.5%)	
Female	8 (38.1%)	13 (56.5%)	
*Residential area (%)*			0.027
Urban	12 (57.1%)	20 (87%)	
Rural	9 (42.9%)	3 (13%)	
*Level of caregivers' education (%)*			0.336
Less than high school	8 (38.1%)	13 (56.4%)	
High school	12 (57.1%)	8 (34.9%)	
Academic	1 (4.8%)	2 (8.7%)	
*Number of children with PKU in the family (%)*			0.626
One patient	18 (85.7%)	20 (87%)	
Two patients	3 (14.3%)	3 (13%)	

Normal ranges of outcomes were determined using reference ranges as follows: 25-hydroxy vitamin D: 20–42 ng/mL; vitamin B12: 200–1900 pg/mL; zinc: 64–140 µg/dL [9]. Reference ranges of phenylalanine, hemoglobin, and hematocrit were defined according to age [23, 24]. The spectrophotometric method was used to measure children's zinc and plasma phenylalanine. Vitamin B12 and 25-hydroxy vitamin D and were determined by the Immunoassay method.

A quantitative process evaluation was conducted to obtain process data on both interventions. This evaluation was conducted using structured interviews that took into account the unique characteristics of each intervention.

### Interventions

#### Educational intervention

The educational intervention was aimed at caregivers; thus children were not involved. The  IMBP was used to guide the development of an educational intervention. We designed intervention activities to alter caregivers' attitudes, subjective norms, self-efficacy, supportive social relationships, and skills using the following behavior change techniques (BCTs): providing information, self-monitoring of behavior, feedback on the outcome of behavior, social support, and problem-solving. Caregivers in the educational group were divided into four groups to facilitate communication and follow-up. For each of these groups, a peer leader was chosen. Group leaders had higher education, good communication skills, and were well-liked by other members. The educational group was offered within the phenylketonuria clinic at the Be'sat hospital in Hamadan and consisted of a combination of in-person and technology-based learning.

Caregivers received two in-person educational sessions. Since the stress associated with change might hinder the transition process, the educational group was led by a PKU specialist in partnership with a clinical psychologist. The psychologist's role was to provide emotional social support to caregivers in order to help them overcome barriers. In the first two-hour session, interactive activities were used to address the following topics: introduction to PKU, the role of diet in controlling PKU, the importance of taking supplements by children, and barriers to dietary management Caregivers received hands-on instruction on how to modify the children's diet during the second three-hour session. We sought to improve caregivers' dietary management skills: the ability to estimate the quantity of phenylalanine in a given amount of food types and the ability to read and use food labels. To do so, caregivers were asked to quantify the phenylalanine level in a given weight of food items, then, the correct amounts were determined through weighing foods. To improve their ability to read food labels, several forms of food labels were presented, and then caregivers were asked to calculate the amount of phenylalanine in food items in particular weight. Caregivers joined a messaging program to receive informational and motivational messages as well as sending daily self-monitoring reports. Caregivers were offered a diary card as a self-monitoring tool and instructed to record estimated phenylalanine values of children's meals. In order to enhance motivation toward dietary management, caregivers were provided tailored feedback messages (through messaging program) in response to the recorded behavior from peer group leaders. Participants were also given educational material, including two leaflets, a reference book, and an educational video on dietary management in PKU and how to prepare low-protein diet foods. Caregivers were encouraged to share these materials with their families.

#### Providing food items

For ten weeks, this intervention included providing free access to five food products. These food items were provided in partnership with a knowledge-based company that specializes in the development of PKU-specific dietary food (Salamat Pazhouhan Sepehr Aria: SPSA). The Food and Drug Administration granted approval to all these food items. The amount of phenylalanine in 100 g of the five food items was as follows: dietary bread: 30 mg, hamburger powder: 150 mg, potato puree: 120 mg, egg powder: 25 mg, and milk powder: 120 mg. A nutrition expert determined the volume needed for each child based on the age group of children and experience of four caregivers. Three volunteer caregivers were asked to assist researchers with the distribution of food items. The caregivers were also given an educational video, a recipe book, and a self-monitoring card. At the 10-week follow-up, the levels of phenylalanine, zinc, 25-hydroxy vitamin D, vitamin B12, hemoglobin, and hematocrit were measured in children of both groups.

#### Statistical analysis

Kolmogorov–Smirnov test was used to check the normality. Baseline comparisons between groups were conducted using t-tests for continuous variables and chi-squared tests for categorical variables. Independent t-test (or ANCOVA) and paired t-test were used to detect differences between the pre-post changes of comparing the level of phenylalanine and micronutrients. Repeated-measurements ANOVA were used to test for differences between two groups in phenylalanine level at the five time-points (i.e., baseline, 10-week follow-up, 6-month follow-up, 12-month follow-up, and 24-month follow up). Generalized estimating equation (GEE) was used to compare the proportion of the children with normal range of phenylalanine level in two groups over time. SPSS 16 software was used for data analysis. The significance level of 5% was considered for all analyses.

## Results

**Fig. 1:**
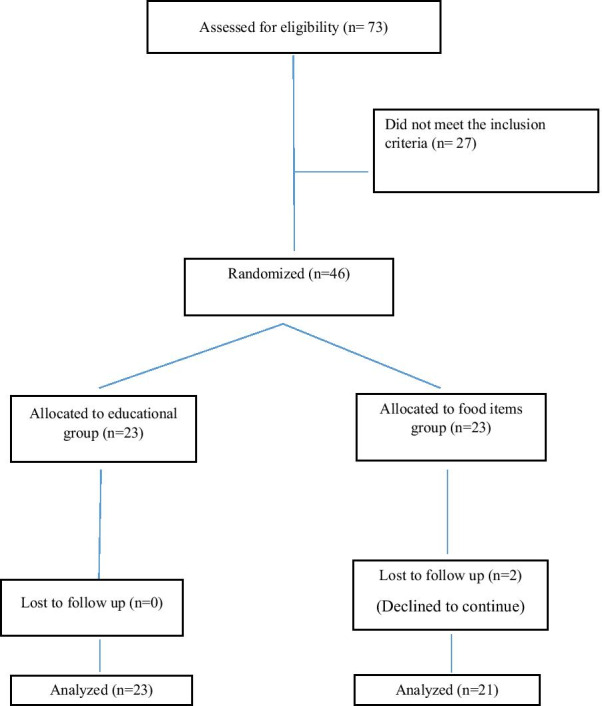
CONSOR diagram

Out of 46 allocated participants, only two from the food items group declined to continue with the study, indicating retention rates were high across both groups (100% and 91% in the educational intervention and providing food items group, respectively) (Fig. 1). Children’s mean age was 6.66 (SD = 3.91) years; mothers and fathers participants’ mean age was 34.72 (SD = 7.52) and 39.96 (SD = 7.60) years, respectively. Of the 44 children, 43 (48%) were female. Around 72% of participants lived in urban areas. The average family size was 4. Forty-seven percent of the caregivers had less than a high school degree, and only about 7% of them had some college degree. There was no difference between the two groups in terms of demographic characteristics, except the residential area (P = 0.002) (Table 1).

Results showed that a large percentage of children had phenylalanine levels out of range indicating non-adherence at baseline: 69.6% and 81% in educational and food items group, respectively.

**Fig. 2:**
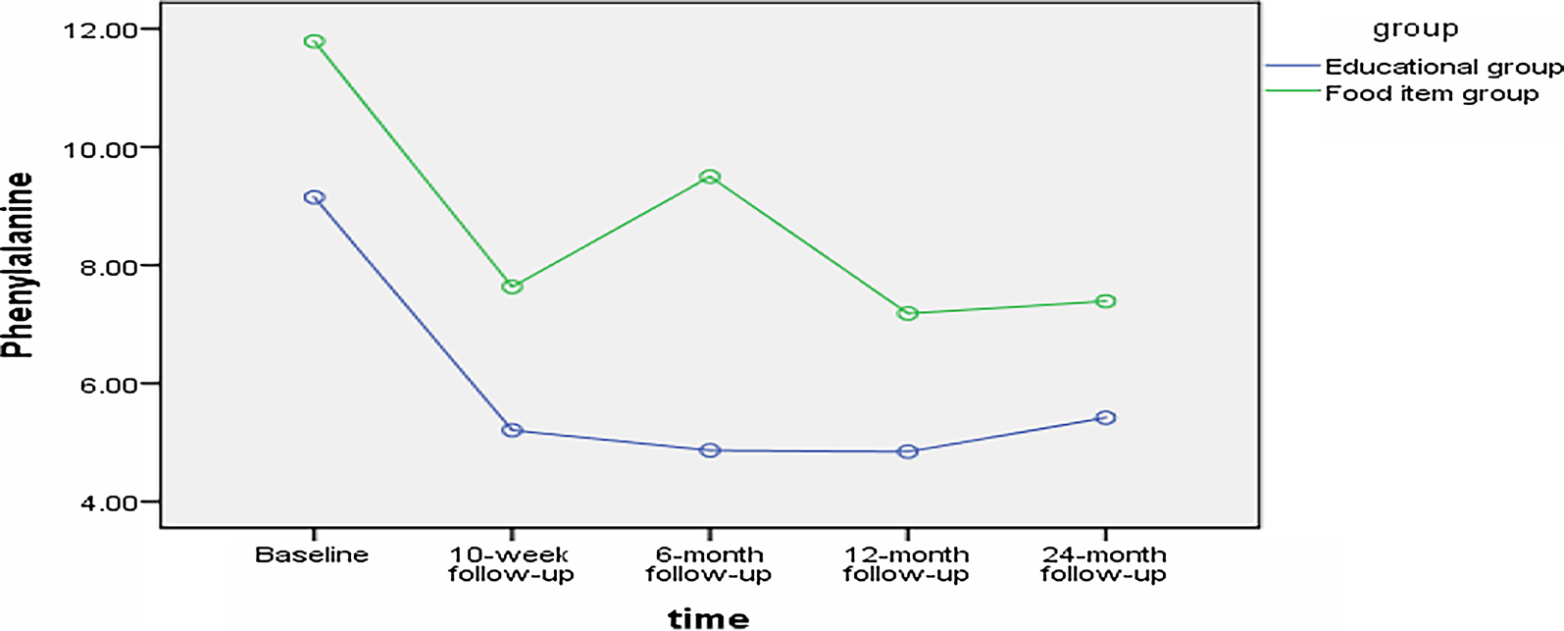
Within-group changes over time for mean of phenylalanine

There were no differences in mean phenylalanine and micronutrients levels between two groups at baseline. (Tables 2, 3). Assessing the effects of interventions showed that the mean of hemoglobin in educational intervention group has been significantly decreased after the intervention (P = 0.041). There was no significant difference in either group in micronutrient level after the intervention (P > 005). The results of paired t-test revealed that both interventions were associated with 10-week reductions in phenylalanine level (P < 0.001) (Table 2). However, covariance analysis showed that there were no significant differences between the two intervention groups at 10-week follow-up in terms of the level of phenylalanine (P > 0.05) indicating the effectiveness of both interventions. Across both groups, the level of phenylalanine decreased significantly from baseline to each follow up interval (Appendix). For example, from baseline to 24-month follow up, both groups decreased the level of phenylalanine (educational group: 8.77 ± 3.91 to 5.33 ± 1.92, P < 0.001) (food items group: 12.20 ± 7.87 to 7.48 ± 3.09, p = 0.002) (Table 3a). While in both groups it was found a significant decrease in phenylalanine overtime, results of repeated-measures ANOVA measuring the change in phenylalanine in groups across five time-points showed that there was a significant difference between groups (F = 4.68, p = 0.03; Table 3(b); Fig. 2).

**Table 2:** Comparison of micronutrient level in groups at baseline and 10-week follow-up

Variable	Educational group (mean ± SD)	Food items group (mean ± SD)	p-value*
*Hemoglobin (g/dl)*			
Baseline	13.29 ± 0.76	13.10 ± 0.96	0.82
10-week follow up	12.95 ± 0.78	12.97 ± 0.66	0.74
p-value**	0.04	0.43	
*Hematocrit (%)*			
Baseline	38.53 ± 1.91	37.87 ± 2.29	0.43
10-week follow up	39.24 ± 2.69	38.48 ± 1.66	0.14
p-value**	0.08	0.18	
*25-hydroxy vitamin D (ng/mL)*			
Baseline	35.90 ± 18.21	38.40 ± 15.34	0.32
10-week follow up	35.68 ± 19.86	30.28 ± 15.14	0.30
p-value**	0.95	0.09	
*Vitamin B12 (pg/ml)*			
Baseline	722.10 ± 341.62	760.00 ± 354.00	0.54
10-week follow up	755.60 ± 428.8	740.30 ± 484.86	0.59
p-value**	0.67	0.75	
*Zinc (µg/dL)*			
Baseline	80.37 ± 19.02	93.58 ± 35.99	0.571
10-week follow up	75.90 ± 37.00	80.25 ± 37.00	0.547
p-value**	0.63	0.33	

*Independent t-test, adjusted for effect of residential area

**Paired t-test

**Table 3:** Results of repeated-measures ANOVAs measuring the change in phenylalanine in groups across time-points

	Educational group (mean ± SD)	Food items group (mean ± SD)	p-value*
*(a)*			
Baseline	8.77 ± 3.91	12.20 ± 7.87	0.23
10-week follow-up	4.90 ± 2.82	7.96 ± 6.68	0.18
6-month follow-up	5.06 ± 1.85	9.27 ± 5.42	0.02
12-month follow-up	4.81 ± 2.34	7.21 ± 3.14	0.02
24-month follow-up	5.33 ± 1.92	7.48 ± 3.09	0.03
p value**	P < 0.001	0.002	

	F statistics	p-value
*(b)*		
Group	4.68	0.036
Time	3.93	0.026
Group × time	1.339	0.268

*ANCOVA Adjusted for the effect of residential area

**Paired t-test: change in phenylalanine from bassline to 24-follow-up

**Fig. 3:**
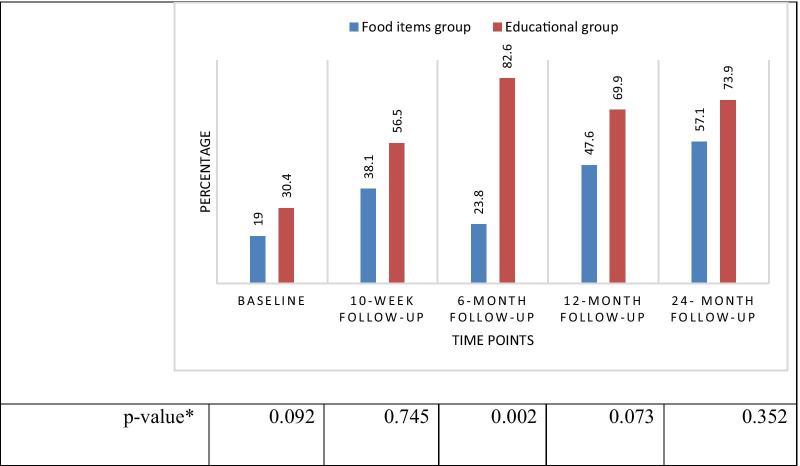
The proportion of the children with normal range of phenylalanine level in two groups over time. *Adjusted for the effect of housing status and residential area

The results of GEE model showed a significant difference between two groups in terms of the proportion of children with a normal level of phenylalanine (P = 0.047). Also, pointwise comparison between two groups showed a statistically significant difference between two groups at 6-month follow up. However, the differences between two groups in other time-points were not statistically significant (Fig. 3).

Process data on educational intervention obtained through questionnaire showed that all of the participants found forming the groups and holding the educational sessions useful, however, about one-third of them were not satisfied with the time of holding the meeting. About 99%, 91%, and 83% of caregivers found the leaflet, self-monitoring cards, and leaflets useful, respectively. Among the aspects of educational intervention, about 40% of the participants did not found the educational video useful. All participants reported that they had received the educational materials. Moreover, discussions and educations provided by clinical psychologists were the most attractive parts of the program.

The results of the process evaluation in providing food items group revealed that all caregivers received food packages and they reported that the distribution of food items as appropriate. Hamburger powder and egg powder had the highest total utilization and hamburger powder was the best food item in terms of taste acceptance by the patients (57.1%). The majority of caregivers reported that they had used the daily nutrition evaluation cards. About half of the participants stated that food items were the same as their children's needs.

## Discussion

This study aimed to compare the effects of two interventions on the level of phenylalanine and micronutrients: educational interventions for caregivers of children with PKU based on IMBP and providing food items. Our result about the large percentage of uncontrolled phenylalanine level is somewhat consistent with other studies conducted in Iran indicating non-adherence [25, 26].

While, the results showed that both interventions were able to significantly reduce the level of phenylalanine, the difference between the two groups was not significant at 10-week follow-up. Interestingly, however, the educational group had a more successful effect in reducing children’s phenylalanine. That is, there was a significant difference between groups in the next follow-ups (i.e., 6-month, 12-month, and 24-month follow-up). Moreover, the results of our study revealed that the percentage of the children within the normal range of phenylalanine was higher in the education group than in providing food items group. In both groups, decrease in phenylalanine level was sustained over time.

Educational and counseling interventions for caregivers include a range of different behavior change techniques (BCTs) from providing mere information to identifying barriers, self-monitoring, providing opportunities for social comparison and environmental changes [27]. According to the IMBP, we developed an educational intervention to help caregivers to identify barriers and helping them to find ways to solution and overcoming barriers to adherence to dietary management, providing social support and reducing stress related to caring for children with PKU, and training on how to adjust the levels of phenylalanine in children’s diets. Previous studies revealed the influence of caregivers' knowledge and skills [14, 19] and the importance of psychological supports such as support groups [28] in increasing the children's adherence to diet and control of phenylalanine. The results of the educational intervention were consistent with the results of the study by Fouad [29].

In some previous studies, it is reported that the interventions based on providing food items and subsidies were effective in improving various aspects of children's food security [For example 12]. The results of our study confirmed that providing food items was effective in reducing the level of phenylalanine. The results of a trial conducted by Concolino et al. showed that following a providing food items intervention, the mean of phenylalanine level was significantly reduced from 15.15 mg/dL to 11.15 mg/dL (P = 0.033) at a 6-month follow-up [30].

A decrease in phenylalanine level was sustained in both groups throughout time; however, the trajectory of these changes was not the same in the two groups. As shown in Fig. 2 while the educational group demonstrated a relatively steady decline in phenylalanine levels, the level of phenylalanine of the food items group children, increased at 6-month follow-up. Given that the providing of food items stopped after 10 weeks, this result was not unexpected. However, during the 6-month and 12-month follow-ups, children's phenylalanine levels dropped significantly, and this trend sustained until the 24-month follow-up. A possible interpretation of this finding could be the onset of symptoms of increased phenylalanine in children, and the successful experience of decreasing phenylalanine following the consumption of low-protein foods, which has led to the provision of these foods by caregivers.

Deficiency in essential micronutrients is one of the common concerns in the diets of patients with PKU. Having to follow a restricted diet in the absence of taking the supplements and formula required by patients makes one susceptible to a reduction of vitamins and minerals [8]. Hence, the effect of both interventions on the levels of micronutrients of the participating children was assessed as a secondary outcome in this study.

Examining the impact of interventions revealed that except for hemoglobin, the micronutrients levels did not change significantly in the two groups. In terms of hemoglobin, all children's hemoglobin levels were within the normal range at baseline and 10-week follow-up, indicating that change in children's hemoglobin was not clinically meaningful. However, the mean of this indicator declined in the educational group from baseline to 1-0-week follow-up. This finding emphasizes the importance of educating caregivers about how to prepare iron-rich foods or iron supplements.

Although it was not statistically significant, the study showed a decrease in the mean of vitamin D3, vitamin B12, and zinc among children in the food item group. A potential explanation for this finding is that children mainly relied on the provided food items to get nutrients and did not get enough nutrients from supplements or other food sources. Since the provided food items were not micronutrient fortified, a reduction in micronutrients was not unexpected. However, determining the percentage of children within a normal range of micronutrients, it was discovered that the majority of children, in both groups, had sufficient micronutrient levels at baseline and 10-week follow-up. In other words, micronutrient deficiencies were not prevalent in the patient cohort (Table 4). This may be attributed to fortification status or adherence to medical formula.

**Table 4:** Number and percentage of children with normal range of micronutrients in groups at baseline and 10-week follows up

	Baseline number (%)	10-week follow up number (%)
*Hemoglobin (g/dl)*		
Educational group	23 (100)	23 (100)
Food items group	21 (100)	21 (100)
*Hematocrit (%)*		
Educational group	23 (100)	23 (100)
Food items group	21 (100)	21 (100)
*25-hydroxy vitamin D (ng/mL)*		
Educational group	18 (78.26)	14 (60.86)
Food items group	14 (66.66)	13 (61.90)
*Vitamin B12 (pg/ml)*		
Educational group	23 (100)	22 (95.65)
Food items group	21 (100)	21 (100)
*Zinc (µg/dL)*		
Educational group	19 (82.60)	14 (60.86)
Food items group	16 (69.56)	14 (66.66)

The process evaluation of interventions showed that the participants were satisfied with both interventions and different parts of the two interventions were used satisfactorily. The positive evaluation of the participants in the section on increasing the skill in food preparation, group discussions as well as understanding the barriers and solutions of dietary management suggests that these strategies be incorporated into the educational programs of caregivers of patients with PKU(Additional file 1).

## Conclusion

The results of our study showed that educational intervention and providing food items were effective in reducing the children’s phenylalanine level; however, the educational group had a more successful effect in reducing children’s phenylalanine. The results of our study suggest that empowering caregivers of patients, creating useful social networks, providing favorable social supports and providing access to special food items may be effective in controlling PKU.

### Supplementary Information

**Additional file 1.** Process evaluation responses from caregivers of intervention groups.

## Data Availability

Patient level data can be made available from the corresponding author after discussion with the trial management committee.

## Appendix

**Table:**

Pairwise comparison: educational group						
Measure: MEASURE_1						
(I) time	(J) time	Mean difference (I–J)	Std. error	Sig.^c^	95% Confidence interval for difference^c^	
					Lower bound	Upper bound
1	2	3.866*	0.493	0.000	2.844	4.888
	3	3.708*	0.642	0.000	2.377	5.038
	4	3.961*	0.674	0.000	2.563	5.360
	5	3.442*	0.560	0.000	2.280	4.605
2	1	− 3.866*	0.493	0.000	− 4.888	− 2.844
	3	− 0.158	0.377	0.679	− 0.940	0.624
	4	0.096	0.467	0.840	− 0.874	1.065
	5	− 0.423	0.410	0.314	− 1.274	0.428
3	1	− 3.708*	0.642	0.000	− 5.038	− 2.377
	2	0.158	0.377	0.679	− 0.624	0.940
	4	0.254	0.406	0.538	− 0.588	1.096
	5	− 0.265	0.341	0.444	− 0.972	0.441
4	1	− 3.961*	0.674	0.000	− 5.360	− 2.563
	2	− 0.096	0.467	0.840	− 1.065	0.874
	3	− 0.254	0.406	0.538	− 1.096	0.588
	5	− 0.519	0.373	0.178	− 1.293	0.255
5	1	− 3.442*	0.560	0.000	− 4.605	− 2.280
	2	0.423	0.410	0.314	− 0.428	1.274
	3	0.265	0.341	0.444	− 0.441	0.972
	4	0.519	0.373	0.178	− 0.255	1.293

Based on estimated marginal means

*The mean difference is significant at the 0.05 level

^a^Group = Educational group

^c^Adjustment for multiple comparisons: Least Significant Difference (equivalent to no adjustments)

**Table:**

Pairwise comparison: food items group						
Measure: MEASURE_1						
(I) time	(J) time	Mean difference (I–J)	Std. error	Sig.^c^	95% Confidence interval for difference^c^	
					Lower bound	Upper bound
1	2	4.237*	0.702	0.000	2.772	5.702
	3	2.919*	1.260	0.031	0.291	5.547
	4	4.980*	1.328	0.001	2.210	7.750
	5	4.711*	1.339	0.002	1.917	7.505
2	1	− 4.237*	0.702	0.000	− 5.702	− 2.772
	3	− 1.318	1.194	0.283	− 3.809	1.172
	4	0.743	1.162	0.530	− 1.681	3.166
	5	0.474	1.202	0.697	− 2.033	2.981
3	1	− 2.919*	1.260	0.031	− 5.547	− 0.291
	2	1.318	1.194	0.283	− 1.172	3.809
	4	2.061*	0.636	0.004	0.735	3.387
	5	1.792*	0.622	0.009	0.494	3.090
4	1	− 4.980*	1.328	0.001	− 7.750	− 2.210
	2	− 0.743	1.162	0.530	− 3.166	1.681
	3	− 2.061*	0.636	0.004	− 3.387	− 0.735
	5	− 0.269	0.268	0.327	− 0.828	0.290
5	1	− 4.711*	1.339	0.002	− 7.505	− 1.917
	2	− 0.474	1.202	0.697	− 2.981	2.033
	3	− 1.792*	0.622	0.009	− 3.090	− 0.494
	4	0.269	0.268	0.327	− 0.290	0.828

Based on estimated marginal means

*The mean difference is significant at the 0.05 level

^a^Group = Food items group

^c^Adjustment for multiple comparisons: Least Significant Difference (equivalent to no adjustments)

## Abbreviations

PKU: Phenylketonuria
IMBP: Integrative Model of Behavioral Prediction
TPB: Theory of Planned Behavior
