# Supplementary material for: Improving phenylalanine and micronutrients status of children with phenylketonuria: a pilot randomized study
Source: Orphanet J Rare Dis. 2021 Nov 12;16:475. doi: 10.1186/s13023-021-02094-8 (PMC8588614; doi:10.1186/s13023-021-02094-8)
Supplement: Supplementary file 1 — Additional file 1. Process evaluation responses from caregivers of intervention groups. [file 13023_2021_2094_MOESM1_ESM.docx]

Results of the process evaluation in the educational group

| no  N(%) | Somewhat  N(%) | yes  N(%) | Question | Row |
| --- | --- | --- | --- | --- |
| 0(0) | 0(0) | 23(100) | Were you satisfied with the training sessions held? | 1 |
| 2(8.7) | 5(21.7) | 16(69.6) | Was the time for the sessions appropriate? | 2 |
| 0(0) | 4(17.4) | 19(82.6) | Were the materials taught in the sessions useful to you? | 3 |
| 0(0) | 7(30.4) | 16(69.6) | Was all the materials you were expected taught? | 4 |
| 0(0) | 0(0) | 23(100) | Did you receive the educational packages (movie-nutrition book-brochure-leaflets-milk formula leaflet-evaluation card-weekly meal plan)؟ | 5 |
| 0(0) | 8(34.8) | 15(65.2) | Did you study brochures and leaflets? | 6 |
| 4(17.4) | 3(13) | 16(69.6) | Did family members and relatives study educational brochures and leaflets? | 7 |
| 4(17.4) | 3(13) | 16(69.6) | Were the brochures and leaflets useful to you? | 8 |
| 9(39.1) | 7(30.4) | 7(30.4) | Did you watch educational videos with your family? | 9 |
| 9(39.1) | 8(34.8) | 6(26.1) | Did the educational videos useful to you? | 10 |
| 2(8.7) | 9(39.1) | 12(52.2) | Did you use evaluation cards? | 11 |
| 6(26.1) | 6(26.1) | 15(65.2) | Was using an evaluation card useful in your patient's food planning? | 12 |
| 2(8.7) | 7(30.4) | 14(60.9) | Did you use the weekly meal plan? | 13 |
| 3(13) | 5(21.7) | 15(65.2) | Was the weekly meal plan useful to you? | 14 |
| 2(8.7) | 3(13) | 18(78.3) | Were you satisfied with your group leader? | 15 |
| 0(0) | 7(30.4) | 16(69.6) | Was the group formed to transfer information useful to you? | 16 |

| **was not attractive(%)** | **was attractive(%)** | **(%) yes** | **Question** |
| --- | --- | --- | --- |
| 8(34.8) | 15(65.2) | Education of nutrition lecturer | 17) Which parts of the program did you like? |
| 1(4.3) | 22(95.7) | Education of psychology lecturer |  |
| 7(30.4) | 16(69.6) | Education of disease lecturer |  |
| 7(30.4) | 16(69.6) | Educational clip inside the session |  |
| 18(78.3) | 5(21.7) | Educational videos |  |
| 20(8.7) | 3(13) | Educational brochures and leaflets |  |
| 19(82.6) | 4(17.4) | Evaluation cards |  |
| 6(26.1) | 17(73.9) | Group discussion |  |
| 17(73.9) | 6(26.1) | Activity within the group and having group leader |  |
| 15(65.2) | 8(34.8) | Question and answer |  |
| 6(26.1) | 17(73.9) | Practical works within the sessions |  |

| Percent | Number of attractive items evaluated | Implemented programs | Question |
| --- | --- | --- | --- |
| 26.1 | 6 | Education of psychology lecturer | 18) Which part of the program was most attractive and effective for you? |
| 4.3 | 1 | Evaluation cards |  |
| 43.5 | 10 | Group discussion |  |
| 4.3 | 1 | Activity within the group and having group leader |  |
| 4.3 | 1 | Question and answer |  |
| 4.3 | 1 | Practical works within the sessions |  |
| 13.0 | 3 | All items |  |
| 100.0 | 23 | Sum |  |

| **Standard deviation** | **mean of score** | **Maximum** | **Minimum** | **Number** | **Question** |
| --- | --- | --- | --- | --- | --- |
| 1.01 | 9.04 | 10 | 7 | 23 | 19) Overall, what score do you give to the program you participated in (from 1 to 10 points) |

Results of the process evaluation in the food items group

| no  N(%) | Somewhat  N(%) | yes  N(%) | Question | Row |
| --- | --- | --- | --- | --- |
| 0(0) | 0(0) | 21(100) | Did you receive the food packages? | 1 |
| 0(0) | 2(9.5) | 19(90.5) | Was the distribution of food appropriate? | 2 |
| 0(0) | 5(23.8) | 16(76.2) | Did you use the dietary bread received? | 3 |
| 1(4.8) | 9(42.9) | 11(52.4) | Was the taste of the dietary bread received acceptable to your child? | 4 |
| 0(0) | 3(14.3) | 18(85.7) | Did you use the dietary hamburger powder received? | 5 |
| 0(0) | 9(42.9) | 12(57.1) | Was the taste of the dietary hamburger powder received acceptable to your child? | 6 |
| 0(0) | 6(28.6) | 15(71.4) | Did you use the dietary mashed potatoes received? | 7 |
| 2(9.5) | 10(47.6) | 9(42.9) | Was the taste of the dietary mashed potatoes received acceptable to your child? | 8 |
| 1(4.8) | 3(14.3) | 17(81) | Did you use the dietary egg powder received? | 9 |
| 2(9.5) | 8(37.1) | 11(52.4) | Was the taste of the dietary egg powder received acceptable to your child? | 10 |
| 2(9.5) | 4(19) | 15(71.4) | Did you use the dietary milk powder received? | 11 |
| 3(14.3) | 7(33.3) | 11(52.4) | Was the taste of the dietary milk powder received acceptable to your child? | 12 |
| 3(14.3) | 5(23.8) | 13(61.9) | Did you use daily meal plan card? | 13 |
| 4(19) | 10(47.6) | 7(33.3) | Was daily meal plan card useful in your patient's food planning? | 14 |
| 2(9.5) | 13(61.9) | 6(28.6) | Did you use the weekly meal plan? | 15 |
| 3(14.3) | 13(61.9) | 5(23.8) | Was the weekly meal plan useful to you? | 16 |

| **Percent** | **positive answer** | **Implemented programs** | **Question** |
| --- | --- | --- | --- |
| 28.6 | 6 | Bread | 17) Which food products did you need the most? |
| 4.8 | 1 | hamburger powder |  |
| 4.8 | 1 | mashed potatoes |  |
| 4.8 | 1 | milk powder |  |
| 57.1 | 12 | All items |  |
| 100.0 | 21 | Sum |  |

| percent | positive answer | Implemented programs | Question |
| --- | --- | --- | --- |
| 4.8 | 1 | bread | 18) Which food products were the least usable to you? |
| 4.8 | 1 | mashed potatoes |  |
| 19.0 | 4 | milk powder |  |
| 4.8 | 1 | All items |  |
| 66.7 | 14 | None |  |
| 100.0 | 21 | Sum |  |

| **Standard deviation** | **mean of score** | **Maximum** | **Minimum** | **Number** | **Question** |
| --- | --- | --- | --- | --- | --- |
| 1.06 | 8.78 | 10 | 7 | 21 | 19) Overall, what score do you give to the program you participated in (Free supply and distribution of food) (from 1 to 10 points) |

(Placeholder1)
